# Novel mutations in *CYBB* Gene Cause X-linked chronic Granulomatous Disease in Pakistani patients

**DOI:** 10.1186/s13052-023-01496-7

**Published:** 2023-08-02

**Authors:** Irum Gul, Taj Ali Khan, Noor ul Akbar, Naila Gul, Rehman Ali, Shahid Niaz Khan

**Affiliations:** 1 Department of Zoology, Kohat University of Science and Technology, 26000 Kohat, Khyber Pakhtunkhwa Pakistan; 2 Institute of Pathology and Diagnostic Medicine, Khyber Medical University, 25160 Peshawar, Pakistan

**Keywords:** Chronic Granulomatous Disease, CYBB gene, Nicotinamide adenine dinucleotide phosphate oxidase

## Abstract

**Background:**

Chronic Granulomatous Disease (CGD) is a primary immunodeficiency that causes susceptibility to recurrent fungal and bacterial infections. The *CYBB* gene encodes gp91^phox^ component of the Phagocytic Nicotinamide adenine dinucleotide phosphate (NADPH) oxidase and specifically, X-linked CGD is caused by mutations in the CYBB gene, located on the X chromosome. The aim of the study was to characterize functional and genetic mutations in X-linked CGD.

**Methods:**

Functional analysis was conducted on the whole blood of seventeen male individuals who were suspected to have X-linked chronic granulomatous disease (CGD). Flow cytometry was employed to assess the capacity of NADPH oxidase, measuring both H_2_O_2_ production and gp91^phox^ protein expression in neutrophils. Additionally, DNA Sanger sequencing was performed for genetic analysis. The pathogenicity of novel mutations was assessed by pathogenicity prediction tools.

**Result:**

Among the seventeen patients evaluated, five patients (P1, P2, P3, P4, and P5) displayed impaired H_2_O_2_ production by their neutrophils upon stimulation with Phorbol myristate acetate (PMA), accompanied by abnormal gp91^phox^ expression. DNA sequencing of the CYBB gene identified specific mutations in each patient. In P1 and P2 (previously reported cases), a hemizygous missense mutation, c.925G > A/p.E309K was identified. In P3 and P4 (novel cases), hemizygous nonsense mutations, c.216T > A/p.C72X were found. Lastly, in P5 (also a novel case), a hemizygous missense mutation, c.732T > G/p.C244W was detected. These mutations reside in exons 9,3 and 7 of the *CYBB* gene, respectively.

**Conclusions:**

The current study contributes to the understanding of the clinical and genetic spectrum associated with X-linked chronic granulomatous disease (CGD). It highlights the significance of early diagnosis in CGD and emphasizes the importance of lifelong prophylaxis to prevent severe infections.

**Supplementary Information:**

The online version contains supplementary material available at 10.1186/s13052-023-01496-7.

## Background

The Phagocytic Nicotinamide adenine dinucleotide phosphate (NADPH) oxidase plays a vital role in eliminating intracellular pathogens by generating microbicidal reactive oxygen species (ROS) [1, 2]. Under resting conditions, NADPH oxidase is comprised of three cytosolic proteins and two membrane proteins, which are encoded by five structural genes (*CYBB, CYBA, NCF1, NCF2, and NCF4*). The membrane proteins, p22^phox,^ and gp91^phox^, are encoded by *CYBA* and *CYBB* genes, respectively. On the other hand, the cytosolic proteins, p47^phox^, p67^phox^, and p40phox, are encoded by *NCF1, NCF2*, and *NCF4* respectively [3, 4]. Upon pathogens stimulation, the cytosolic proteins (p47phox, p67phox, and p40phox) associated with RAC2 combine with p22^phox^ and gp91^phox^ to form an active NADPH oxidase complex that produces ROS [5].

Mutations in the five structural genes (*NCF1, NCF2, NCF4, CYBA, CYBB*) are associated with chronic granulomatous disease (CGD), a condition characterized by impaired production of reactive oxygen species (ROS) [2]. In CGD, the defective phagocytic NADPH oxidase-killing ability of pathogens results in chronic fungal and bacterial infections, leading to the formation of characteristic granulomas, a hallmark of the disease [3]. Notably, recurrent life-threatening infections in CGD are often caused by pathogens such as *S. aureus*, *Aspergillus* species, *Salmonella* species, Klebsiella species, *mycobacteria* spp., and *Burkholderia cepacia* [6].

CGD was first identified as an X-linked/autosomal recessive disease in early 1950s [4]. Among the various forms of CGD, the X-linked form of CGD is the most common (∼ 65%) and is caused by mutations in the *CYBB* gene positioned on the X-Chromosome. This gene encodes the gp91^phox^ protein, also known as NOX2 [7]. Autosomal recessive CGD (ARCGD) results from mutations in *CYBA* (∼ 5%), *NCF1*(∼ 20% ), *NCF2* (∼ 5%), or *NCF4* (1%) genes [6, 8]. Three variants of X-linked CGD have been characterized so far: X91^0^(complete absence of gp91^phox^ expression and NADPH oxidase activity), X91^−^( reduced gp91^phox^ expression and NADPH oxidase activity), and X91^+^(normal gp91^phox^ expression with residual NADPH oxidase activity first). These variants resulting from heterogenous hemizygous mutations that differently affecting the function and expression of gp91^phox^ protein [9].

Lack of medical knowledge, limited awareness about CGD, and the absence of diagnostic centers in many countries, including Pakistan, contribute to the delay in diagnosing CGD [10, 11]. Early and accurate diagnosis of CGD, particularly X-linked CGD, is crucial for the prognosis of affected individuals. Any delay in diagnosis hinders the timely initiation of specific treatments for CGD patients, leading to poor prognosis and an increased risk of mortality in these individuals.

In Pakistan, physicians rarely consider genetic diagnosis for CGD, and diagnosis primarily relies on clinical phenotypes. However, the clinical overlap among different conditions often makes it challenging to establish a definitive diagnosis. While there is extensive research on the molecular diagnosis of CGD patients globally, limited information is available regarding the molecular diagnosis of CGD patients in Pakistan. therefore, the purpose of our study was to investigate and characterize genetic alterations in *CYBB* gene in Pakistani patients using biochemical and genetic analyses.

## Methods

### Ethical statements and patients

This study included seventeen male patients with strong suspicion of X-linked chronic granulomatous disease (XCGD). All patients were confirmed to be HIV-negative. The collection of blood samples followed the approved protocol after taking consent from either the patients themselves or their parents. Blood samples were obtained from both the patients and control subjects. The collected blood samples were used for functional assessment as well as genetic analysis. This study has obtained approval from the ethical committee of Kohat University of Science and technology (KUST), Pakistan.

### Dihydrorhodamine- 123 assay for quantification of H_2_O_2_

The oxidative burst, specifically the production of H_2_O_2_, in neutrophils was analyzed following a previously published protocol [12]. Briefly, a total of 5 ml of blood was collected from both healthy controls and patients in heparinized tubes. The whole blood samples were then stimulated with phorbol myristate acetic acid (PMA) at a concentration of 300 ng/ml for a duration of 30 min. Following this, the samples were incubated with the dihydroergotamine (DHR) 123 assay at 37^0^ C for 20 min. The Whole blood was then lysed using RBC lysis solution at room temperature, and the cells were subjected to two washes with PBS. subsequently, the cells were fixed using a 2% paraformaldehyde solution. Flow cytometry analysis was performed using a BD FACSCanto II flow cytometer. The data obtained were analyzed using FlowJo software (Treestar, Inc, Ashland, Ore).

### Flow cytometric analysis of NADPH oxidase complex

Flow cytometric analysis was performed to evaluate the expression of NADPH oxidase protein (gp91^phox^),as previously described [13, 14]. To specifically identify the membrane-associated NADPH oxidase (gp91^phox^) protein, neutrophils were incubated with anti-gp91^phox^ antibody and subsequently washed twice with PBS. The fixation process was carried out in PBS comprising 1% paraformaldehyde, and the analysis was performed using a BD FACSCanto II flow cytometer. To identify the components of cytoplasmic proteins, the cells were permeabilized and fixed using the Cytoperm/Cytofix Plus kit, as per the manufacturer’s instructions. Subsequently, the cells were stained for 30 min at 4 °C with anti-p40^phox^, anti-p47^phox^, and anti-p67^phox^ antibodies. After two washes with PBS, the cells were analyzed using a BD FACSCanto II flow cytometer. Data analysis was conducted using FlowJo software (Treestar, Inc, Ashland, Ore).

### CYBB gene sequence analysis

Genomic DNA (gDNA) was extracted from ethylenediaminetetraacetic acid (EDTA)-chelated blood samples using the Wizard® Genomic DNA Purification kit from Promega. Polymerase chain reaction (PCR) was performed to amplify a total of 13 exons of CYBB, including exon-intron boundaries. The DYEnamic ET Terminator Cycle Sequencing Kit was utilized to sequence the PCR amplified products using the MegaBACE 1000 sequencer.

### Assessment of pathogenic genetic variations in CYBB gene

Pathogenicity-predicating tools such as Polyphen-2, SIFT, and Mutation Taster were utilized to evaluate the mutation pathogenicity [15–17].

### Evaluation of multiple sequence alignment and 3D structure

Sequences of gp91^phox^ from various organisms were obtained from the protein database (www.uniprot.org) and was multiple-aligned with gp91^phox^ mutants employing clustal X [18]. Comparative structure of full-length human gp91^phox^ (with a missing sequence of 200–280 amino acids) were constructed through program MODELLER 9v19 [18] while taking gp91^phox^ from Cylindrospermum stagnale and human as template (PDB id: 5O0T, 5O0X and 3A1F) [19]. The Output of MODELLER contained 3D model for the target sequence (wild type and mutants of gp9^phox^) were used with the entire non-hydrogen side as well as main chain atoms. We have developed various comparative models for every wild type and mutants gp91^phox^ and the PROCHECK and ProSA programs were utilized to choose the best structure [20].

### Evaluation of *M. tuberculosis* growth in monocytes derived macrophages

We further assessed the phagocytic capacity of monocyte-derived macrophages (MDMs) from both NOX2- deficient patients and control. We also examined their ability to control the growth of *M. tuberculosis* in response to in vitro treatment with recombinant human interferon gamma (rhIFN), following the previously described protocol [21].

### Statistical analysis

Statistical analysis was conducted using GraphPad PRISM 5.00 software from GraphPad Software (San Diego, CA, USA). An unpaired t-test was employed for the analysis, and a significant level of P < 0.05 was used to determine statistical significance.

## Results

### Clinical presentations in X-linked CGD patients

**Fig. 1:**
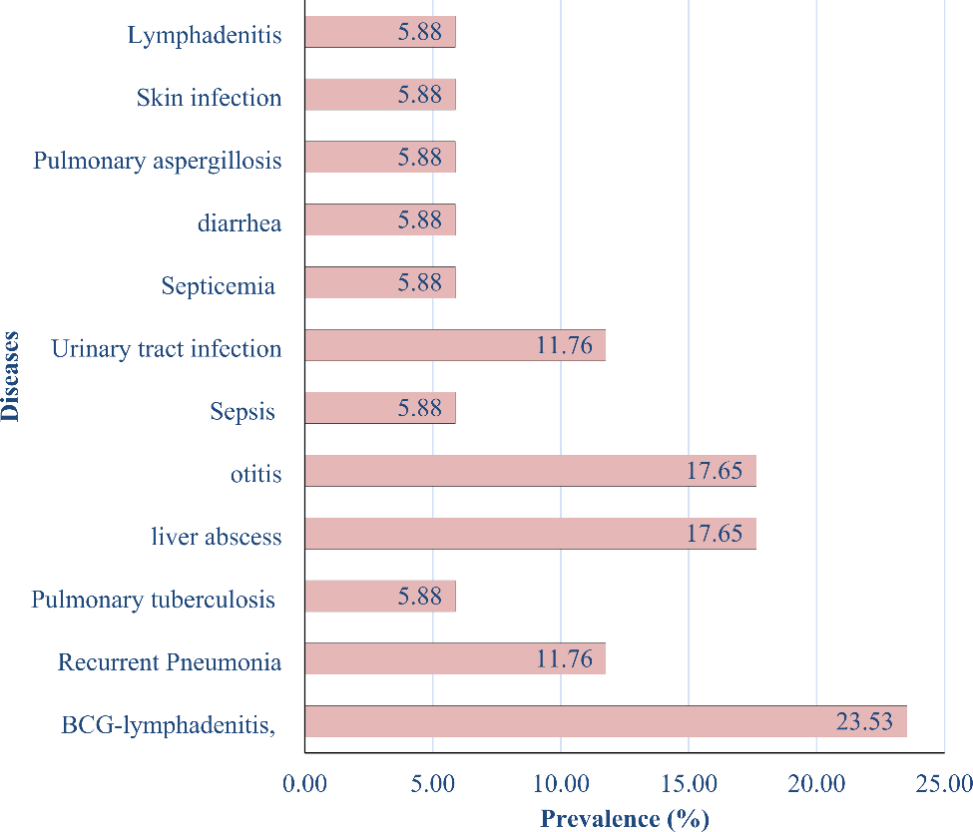
Map of study areas, 4 CGD patients belongs to district Mardan, 8 were from district Peshawar, 3 from district Kohat and 2 from district Karak. Percentage load of infections in clinically suspected patients

Among the Pakistani XCGD patients (P1, P2, P3, P4, and P5), a higher frequency of BCG complications (23.53%) was observed. Other clinical presentations included Liver abscess and Otitis media (11.65%), Urinary tract infection and Pneumonia (11.76%), diarrhea, Tuberculosis (TB), Septicemia, Pulmonary aspergillosis, Skin infection, and Lymphadenitis 5.88% each (Fig. 1).

### The X-linked CGD patients displayed impaired oxidative burst and NOX2 expression

**Fig. 2:**
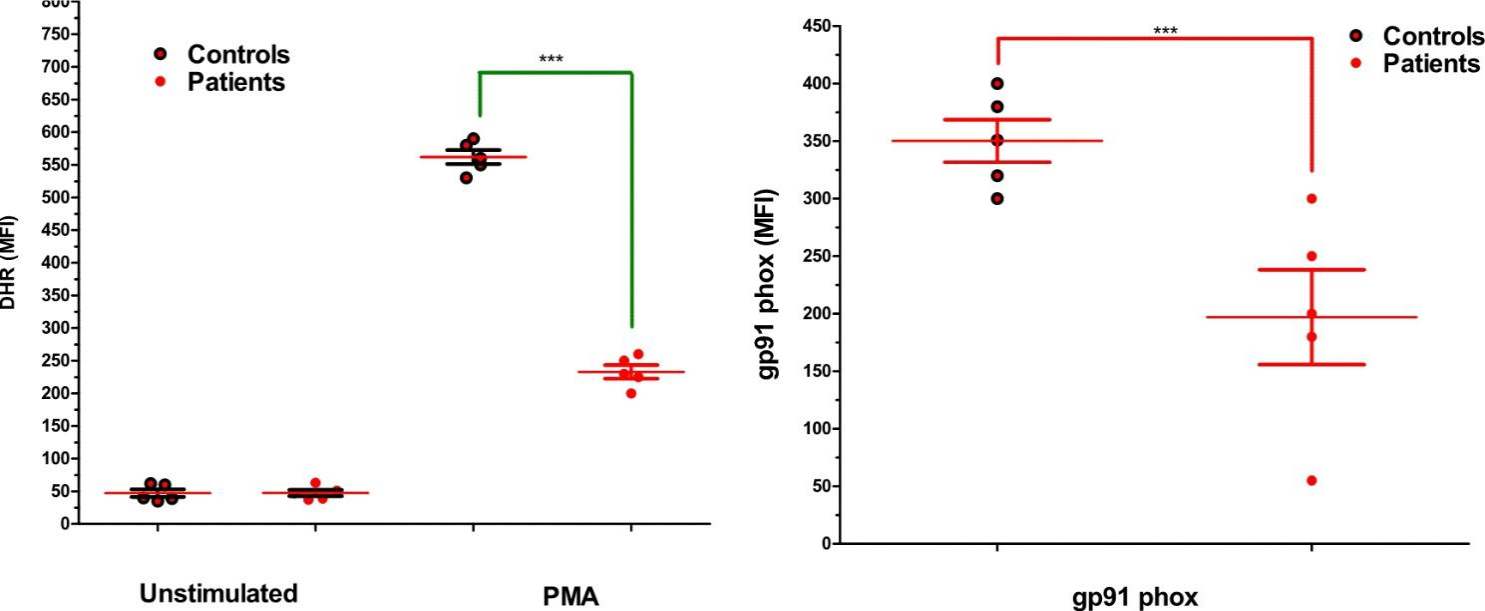
(**A**) show reduced H2O2 by NADPH oxidase of patients (P1, P2, P3, P4 and P5) in comparison to healthy controls, similarly (**B**) flow cytometric analysis of gp91^phox^ in neutrophils of P1, P2, P3, P4, and P5 reveal aberrant expression respectively. The unfilled circles showing the control group of the same ages while the black filled circle representing the XCGD patients

The DHR-123 test was conducted to measure H_2_O_2_ level in 17 male CGD patients. Among them, five patients (P1, P2, P3, P4 and P5) exhibited reduced H_2_O_2_ production in their neutrophils (Fig. 2). Flow cytometric analysis was conducted to evaluate the expression of the gp91^phox^ component of NADPH oxidase. The results indicated that only two patients (P3 and P4) had the X91^0^ CGD variant, characterized by the absence of gp91^phox^ expression and the complete loss of NADPH oxidase activity. On the other hand, three patients (P1, P2 and P5) exhibited the X91-CGD variant, characterized by low levels of gp91^phox^ expression and a corresponding reduction in H_2_O_2_ production compared to healthy controls. It should be noted that the presented data in Fig. 2B only represents the NADPH expression (gp91^phox^) of the genetically characterized patients, while the other components of NADPH oxidase (p22^phox^, p47^phox^, p67^phox^, and p40^phox^) were found to be normal (Data not shown).

### Genetic analysis of XCGD patients revealed heterogeneous variations in the CYBB gene

**Fig. 3:**
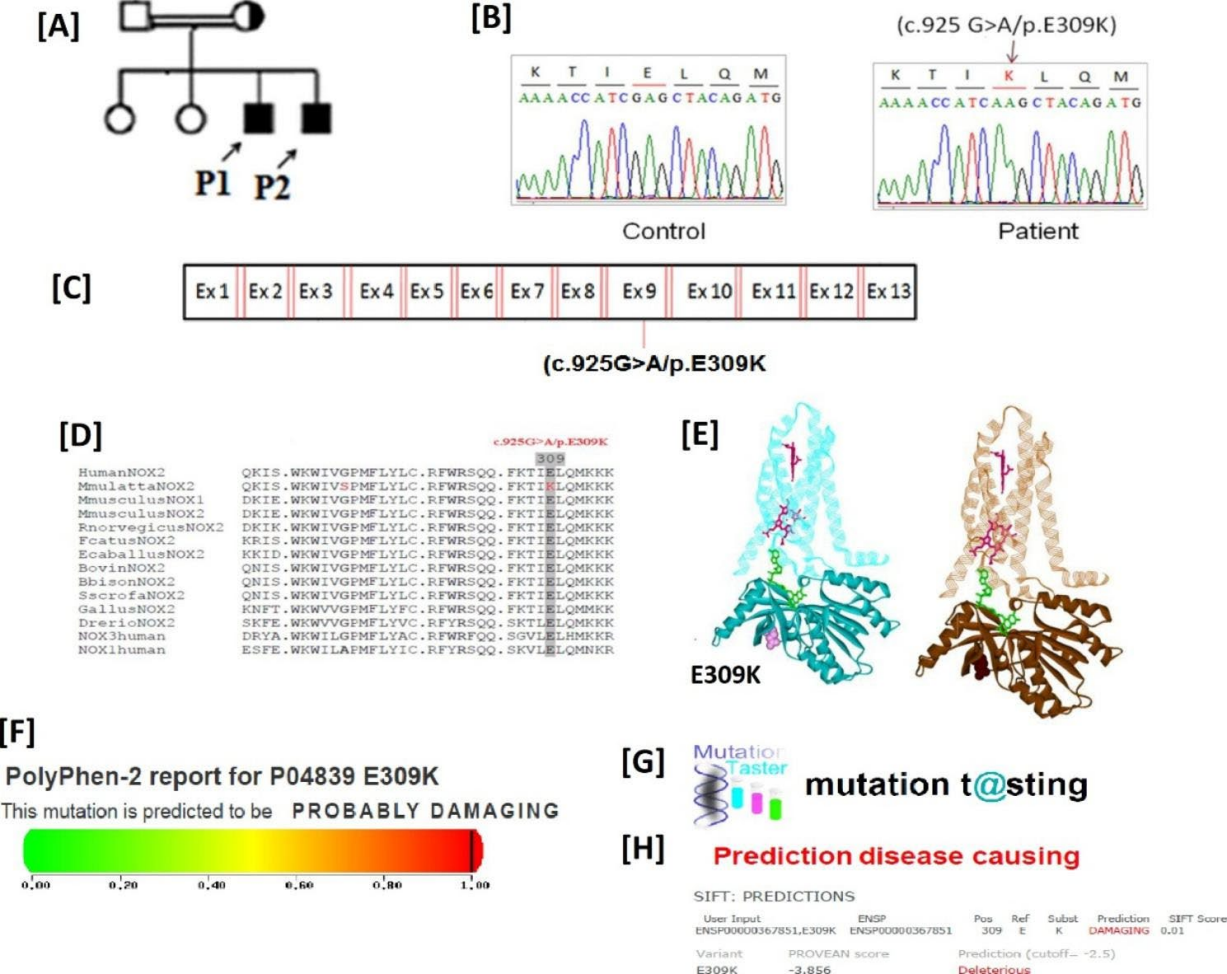
(**A**), (**B**), and (**C**) showing family pedigree of P1/P2 chromatogram and location of mutation (c.925G > A/p. E309K) in exon 9 respectively while (**D**) and (**E**) representing residue p. E309 across the species and 3D structure analysis of wild and mutant type gp91^phox^ respectively, (**F**), (**G**) and (**H**) displaying the prediction tools (Polyphen-2, Mutation Taster and SIFT). The black filled squares represent patients

**Fig. 4:**
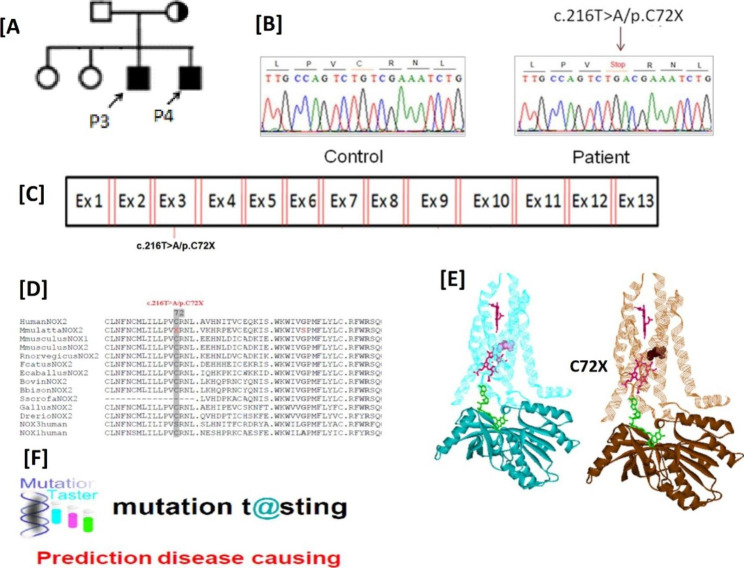
(**A**), (**B**), and (**C**) showing family pedigree of P2/P3, chromatogram and location of mutation (c.216T > A/p.C72X) in exon 3 respectively while (**D**) and (**E**) indicating residue p.C72 across the species and 3D structure analysis of wild and mutant type p.C72X gp91^phox^ respectively. (**F**) indicating the Mutation Taster result. The black filled squares represent patients

**Fig. 5:**
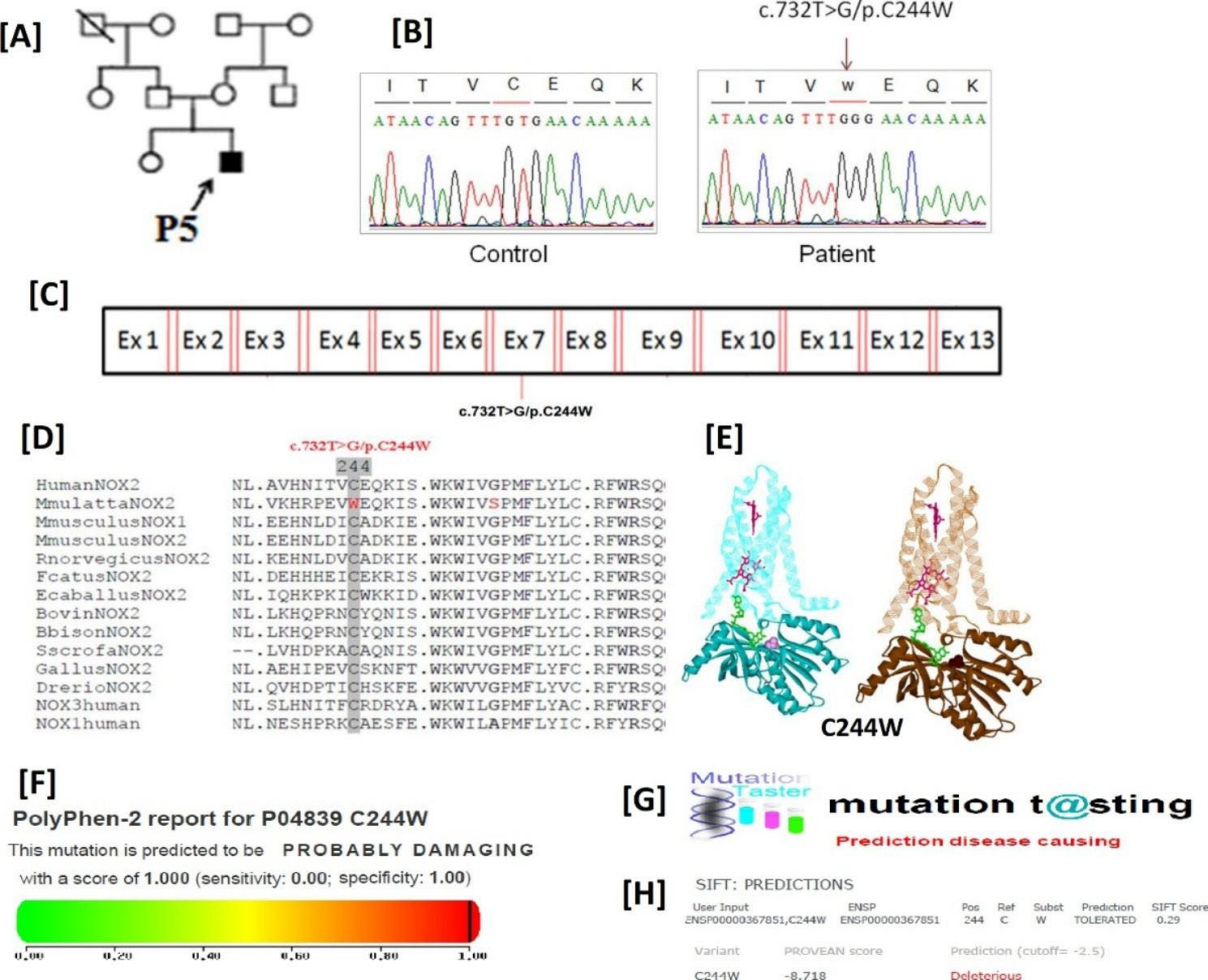
(**A**), (**B**), and (C) presenting family pedigree of P5, chromatogram and location of mutation (c.732T > G/p.C244W) in exon 7 respectively while (**D**) and (**E**) representing residue p.C244W across the species and 3D structure analysis of wild and mutant type p.C244W gp91^phox^ respectively, (**F**), (**G**), and (**H**) representing the prediction tools (Polyphen-2, Mutation Taster and SIFT). The black filled squares represent patients

After suspecting X-linked CGD in male patients with abolished H_2_O_2_ production, we conducted genetic analysis to investigate possible CYBB gene alterations. The hemizygous mutations were identified in the CYBB gene through genetic analysis. Among the patients, three individuals (P1/P2 with c.925G > A/p. E309K mutation, and P5 with c.732T > G/p.C244W mutation demonstrated missense mutations, while 2 patients (P3 and P4) exhibited nonsense mutations (c.216T > A/p.C72X) in the CYBB gene (Table 1; Figs. 3, 4 and 5).These identified mutations were found to be located in in exon 3, exon 7, and exon 9 of the CYBB gene (Figs. 3C, 4 and 5 C). It is worth mentioning that missense mutation (c.925G > A/p. E309K) has been previously reported [22], while based on our search, novel genetic variations (c.925G > A/p and c.732T > G/p.C244W) were not found in genetic variation databases like ExAC, LSDB, 1000G, Ensemble, or CYBB mutation database, indicating that these mutations are novel.

**Table 1:** Summary of Mutational analyses of X-linked CGD patients

Patients	CGD variant	Mutation	Position	Bioinformatic pathogenicity				Reference
				Mutation Taster	Polyphen2	SIFT	Proven	
				Disease causing	Damaging	Damaging	Deleterious	
P1*	X91^−^	c.925G > A/p.E309K	Exon 9	+	+	+	+	[22, 23]
P2*	X91^−^	c.925G > A/p.E309K	Exon 9	+	+	+	+	[22, 23]
P3*	X91^o^	c.216T > A/p.C72X	Exon 3	+	NA	NA	NA	Novel
P4*	X91^o^	c.216T > A/p.C72X	Exon 3	+	NA	NA	NA	Novel
P5	X91^−^	c.732T > G/p.C244W	Exon 7	+	+	+	-	Novel

Symbols: *(brother), + (pathogenic), - (not pathogenic), NA (not applicable), X91^−^(reduced), X91^o^ (absent)

### *In Silico* analyses predicting that sequence variations affect the NAPDH oxidase activity

The multiple alignments of the identified mutations i.e.C244W, E309K, and C72X revealed that they are in highly conserved motifs of the NOXs (Figs. 3D, 4D and 5D). This suggests that these mutations may have an impact on the function of gp91^phox^. The mutations such as E309K are residing in the FAD binding pocket, which could disrupt the FAD binding to the gp91^phox^ and hence its functionality. Additionally, the nonsense mutation C72X lead to a truncated protein which may completely abolished NADPH oxidase activity. Computational models of gp91^phox^ further support our functional and genetic analyses, showing the presence of a truncated protein (Figs. 3E, 4E and 5E).

### Mutation pathogenicity tools evaluation suggesting that sequence alterations are pathogenic

To assess the pathogenicity of the identified mutations, the we utilized mutation prediction tools like Polyphen-2, Mutation Taster and SIFT. The results from these tools consistently indicated that the sequence variations found in our study are disease-causing and thus pathogenic, as shown in Fig. 3 (F), Fig. 4 (F-G), and Fig. 5 (F-G). The genetic variations observed in the CYBB gene are strongly associated with the presence of CGD, other possibilities cannot be completely ruled out at this time, such as the presence of a genetic mutation in a gene not yet linked to CGD. Further investigation is needed to gain a comprehensive understanding of the underlying mechanisms and potential contributions of other factors.

### rhIFN-γ improved the phagocytic activity of monocytes derived macrophages

**Fig. 6:**
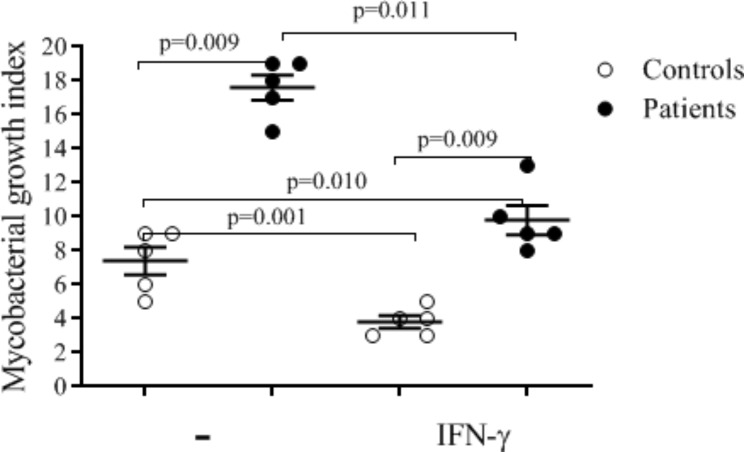
Defective control of the proliferation of *M. tuberculosis* by MDMs from gp91^phox^ functional deficient patients is improved by rhIFN-γ. The results of untreated (-) and rhIFN-γ treated (rhIFN-γ) MDMs are shown (**A**) Phagocytosis, (**B**) *M. tuberculosis* growth index. The bacterial proliferation index was determined based on the ratio of the CFU numbers on day 6 to the CFU number on day 0 after the challenge (Control N = 5 and Patients N = 5). **-** indicating without rhIFN-γ treatment of MDMs

The study findings indicate that MDMs from patients with functional deficiency of gp91^phox^ exhibit impaired control over the proliferation of *Mycobacterium tuberculosis* (*M. tuberculosis*). However, when these MDMs were treated with recombinant human interferon (rhIFN-γ), the MDMs derived from patients with gp91^phox^ functional deficiency exhibited improved capability in controlling the proliferation of *M. tuberculosis* compared to MDMs from healthy controls (Fig. 6). This finding suggests that the administration of interferon gamma may have a positive impact on enhancing the immune response against *M. tuberculosis* in patients with gp91^phox^ functional deficiency (Fig. 6). It is worth mentioning that our this experiment needs further validation using more rhIFN-γ treated MDMs from X-linked CGD patients with similar underlying mutations to characterize the exhibited improved capability in controlling the proliferation of *M. tuberculosis*.

## Discussion

We conducted an analysis of underlying mutations, specifically (c.925G > A/ p.E309K) in exon number 9, (c.216T > A/p.C72X) in exon 3, (c.732T > G/p.C244W) in exon 7, within the CYBB gene. These mutations were observed in individuals who exhibited various clinical manifestations, notably severe bacterial and fungal infections, including mycobacteriosis. Our investigation encompassed genomic, *in silico*, and functional analyses, which indicated that these novel genetic variants contribute to the pathogenesis process by potentially affecting the NADPH oxidase activity. The average age of the XCGD patients in our study was 9 years old at the time of diagnosis. Our data suggest that the residues involved in the sequence variations are highly conserved across different species and are typically absent in healthy individuals within the same population as the patients. Nonetheless, further research is necessary to establish a connection between these novel mutations and the mechanisms underlying pathogenesis, particularly in relation to the abnormal function of NADPH oxidase.

The clinical features of X-linked NOX2-deficient CGD are presented with earlier onset of recurrent microbial and fungal infections and aberrant inflammation leading to early identification of the disease during childhood [24, 25]. In accordance with this, the patients in our study established characteristic CGD phenotypes during infancy. In various cases of gp91^phox^ deficiency, the mycobacterial infections like tuberculosis have been reported [3, 26]. Importantly, the immunization of newborns in Pakistan are mandatory with BCG at birth against the protection of TB. In fact, one of our patients exhibited adverse BCG vaccination reaction and later developed tuberculosis. BCGitis occurred in 23.53% of our patients with tuberculosis, which is almost similar to the other published data [27, 28] and lower than Iranian patients (56%) [29]. It is presumed that such differences could be due to the type of BCG strain, age at the time of vaccination, amount of BCG dosages and type of mutations. Pulmonary infections were the common clinical phenotype of XCGD in our patients, which is in accordance with previous reports [8, 24, 29, 30].

*Staphylococci* and *Aspergillus* are the main causal agents found in CGD [31], which were also the causal pathogens in some of our CGD patients. In various countries, the limited medical awareness about primary immunodeficiencies (PID) is the most apparent reason of late diagnosis of the disease [10, 11, 32–34]. Globally, the average age of diagnosis for patients with XCGD is 5 years [25], in contrast to this, our cohort exhibited a slightly higher mean age of diagnosis, which was 9 years, suggesting late diagnosis of CGD in Pakistan. Other factors may also contribute to the late identification of the CGD in our patients since clinicians in Pakistan tend to focus on signs and symptoms (e.g., diarrhea, sinusitis, TB, pneumonia, and bronchitis) rather than the underlying PID.

Various subtypes of X-linked chronic granulomatous disease (XCGD) can arise from different types of mutations in the CYBB gene. The specific mutations can give rise to distinct variants of XCGD with varying clinical presentations and severity [32]. In our cohort of XCGD patients, mutations in *CYBB* gene led to the two variants of XCGD (X91^o^ and X91^−^). Similarly, missense mutations in the CYBB gene of P1, 2 and P5, caused low expression of NOX2 (X91^−^ variant) with impaired activity of NADPH oxidase, while nonsense mutations in CYBB gene of P3 and P4 resulted in the absence of NOX2 expression (X91^0^ variant).The identification of mutations: c.925G > A/p.E309K in our two siblings (P1/P2) has been previously reported to affect both ROS production and gp91^phox^ expression, as in accordance with [22, 23]. Most reported mutations are found throughout the 13 exons of the *CYBB* gene [35].

Accordingly, we identified the mutations, (c.216T > A/p.C72X, c.216T > A/p.C72X, c.925G > A/ p.E309K and c.732T > G/p.C244W) which are residing in exon 3, exon 7, and exon 9 of CYBB gene respectively. Our in silico and functional analyses suggest that these novel mutations (c.216T > A/p.C72X, c.216T > A/p.C72X, and c.732T > G/p.C244W) affect the respiratory burst of NADPH oxidase. However, transgenic experiments are necessary to determine the impact of these new variants on the assembly of NOX2 with other proteins and the functions of NADPH oxidase. Bionda and her colleagues employed a similar strategy to effectively exploit missense mutations in the CYBB gene [36]. However, in the case of our CGD patients, rhIFN-𝛾 was not used as an immuno adjuvant. Treatment options for CGD typically include antibacterial prophylaxis (trimethoprim-sulfamethoxazole), antifungal prophylaxis (itraconazole), recombinant human interferon gamma, (rhIFN) and Hematopoietic stem cell transplant (HSCT). All these have been shown to aid in the recovery from infections in CGD [37–42]. In our cohort, CGD patients utilized antibacterial and antifungal prophylaxis, but they did not receive rhIFN as immuno adjuvant nor undergo HSCT. However, in vitro treatment of MDMs from gp91^phox^ deficient patients with rhIFN-𝛾 improved their ability to control growth of *M. tuberculosis*, which aligns with previous reports [43, 44]. Importantly, we suggest that further validation of our result regarding the in vitro treatment of rhIFN-𝛾 should involve more MDMs from gp91^phox^ functional deficient patients with similar underlying mutations.

## Conclusion

We conclude that mutations in the *CYBB* gene cause X-linked CGD, and thus expanding the clinical and genetic spectrum of the disease. Additionally, early diagnosis of CGD is crucial for timely intervention, enabling physicians to treat infections before they escalate to a severe life-threatening stage.

## Electronic supplementary material

Below is the link to the electronic supplementary material.

Supplementary Material 1

## Data Availability

The datasets used and/or analyzed during the current study available from the corresponding author on reasonable request.

## Declarations

**Ethics approval and consent to participate**.

The experimental protocols were approved by the ethical committee of Kohat University of Science and Technology (KUST) under reference number (KUST/Ethical committee/18 − 2). All methods were performed in accordance with the ethical standards as laid down in the Declaration of Helsinki and its later amendments. Written informed consent was obtained from all participants.

**Consent for publication**.

Written informed consent for publication was obtained.

## Abbreviations

CGD: chronic granulomatous disease
*CYBB*: Cytochrome B-245 beta chain
DHR: dihydrorhodamine
HEC: higher education commission
HIV: human immunodeficiency virus
H_2_O_2_: hydrogen peroxide
NADPH: oxidase nicotinamide adenine dinucleotide phosphate oxidase
PBS: phosphate buffered saline
PCR: Polymerase Chain Reaction
PID: primary immunodeficiencies
PMA: phorbol myristate acetic acid
ROS: reactive oxygen species
SIFT: sorting intolerant from tolerant
XCGD: X-linked chronic granulomatous disease
