# Supplementary material for: Novel mutations in CYBB Gene Cause X-linked chronic Granulomatous Disease in Pakistani patients
Source: Ital J Pediatr. 2023 Aug 2;49:95. doi: 10.1186/s13052-023-01496-7 (PMC10399011; doi:10.1186/s13052-023-01496-7)
Supplement: Supplementary file 1 — Supplementary Material 1 [file 13052_2023_1496_MOESM1_ESM.docx]

Subject: Response to Revision Requests - Manuscript [[ITJP-D-22-00455](blob:https://web.whatsapp.com/145cc55f-c781-45f0-a78d-bdccdd214083)]

Dear Editor,

We hope this email finds you well. We would like to express our gratitude for the thorough review of our manuscript entitled "Mutations in CYBB Gene Caused X-linked Chronic Granulomatous Disease in Pakistani Patients." We appreciate the valuable feedback provided by the reviewers, which has significantly contributed to improving the quality and clarity of our work.

We have carefully considered the reviewers' comments and have made the necessary revisions to address their suggestions. We have also incorporated the requested changes pointed out by the editor. In this email, we would like to present our detailed responses to each of the revision points.

1. Mismatch Primary Title: We acknowledge the discrepancy in the primary article title between the manuscript and the submission system. We have revised the title according to the suggestion of Reviewer 1 to better reflect the unique aspects of our study. The updated title is now "Novel Mutations in CYBB Gene Caused X-linked Chronic Granulomatous Disease in Pakistani Patients."
2. Mismatch Order of Authors: Upon reviewing the order of authors, we realized that an error occurred due to our negligence. We apologize for the confusion caused by this oversight. We would like to maintain the sequence of authors as follows: Irum Gul, PhD; Taj Ali Khan, PhD; Noor ul Akbar, PhD; Naila Gul, PhD; Rehman Ali, PhD; Shahid Niaz Khan, PhD. We apologize for any inconvenience caused and assure you that the order of authors has been corrected accordingly.
3. Corresponding Author: We appreciate the editor's request to clearly indicate the corresponding author's details in the manuscript file. We apologize for the initial omission. As per your instructions, we have now included the following information after the authors' affiliations:

Corresponding author: Name: Shahid Niaz Khan

Email: [shahid@kust.edu.pk](mailto:shahid@kust.edu.pk)

Affiliation: Department of Zoology, Kohat University of Science and Technology

City: Kohat

Country: Pakistan

4. Missing Affiliation 2: We apologize for the missing affiliation 2 in our manuscript. The correct affiliation 2 is as follows:

Name: Taj Ali Khan

Email: [tajalikhan.ibms@kmu.edu.pk](mailto:tajalikhan.ibms@kmu.edu.pk)

City: Peshawar

Country: Pakistan

We have now included the missing affiliation, ensuring that it aligns with the author's list.

5. Mismatch Primary Abstract: Taking into account the comments from Reviewer 1 and Reviewer 2, we have revised the abstract to address the concerns raised. Specifically, we have reformulated the results section of the abstract as suggested by Reviewer 1, and we have extensively revised the English language quality of the abstract as recommended by Reviewer 2. The revised abstract has been submitted along with this response.
6. Missing Initials and Contribution for Rehman Ali: We apologize for the oversight regarding the missing initials and contribution for Rehman Ali in the "Authors' contributions" subsection. We have included the initials (RA) and the corresponding contribution for Rehman Ali as per your instructions.
7. Missing "Declarations" Heading and Incomplete Subsections: We regret the absence of the "Declarations" heading and the incomplete subsections in our manuscript. We have rectified this by adding the "Declarations" heading before the declaration section, and we have provided the complete subsections as requested.

**Note:** "We have carefully revised the manuscript according to the reviewers' suggestions, incorporating all the necessary changes. To facilitate the identification of revisions, we have highlighted all the modifications made in the revised manuscript using red text. These highlights will allow the reviewers and editor to easily locate and assess the changes. We believe that these modifications have significantly improved the clarity and scientific rigor of our work."

Additionally, we kindly request your assistance in expediting the review process for our revised manuscript. Meeting the graduation requirement within the specified timeframe is of utmost importance, and an expedited review would greatly facilitate timely completion.

We would like to express our sincere appreciation for your guidance throughout this review process.

Regards,

Professor Dr. Shahid Niaz Khan

Dean Faculty of Biological Sciences

Kohat University of Science and Technology, Kohat

**Response letter 2 with revised English**

Dear Carlo Caffarelli,

Editor-in-Chief,

Italian Journal of Pediatrics.

**Subject: Revision requested for Italian Journal of Pediatrics - ITJP-D-22-00455**

We are submitting the second revised version of the manuscript titled "Novel Mutations in the CYBB Gene Causing X-linked Chronic Granulomatous Disease in Pakistani Patients" for consideration of publication in the Italian Journal of Pediatrics.

We are very much grateful to the editor, as well as the reviewers, for their valuable comments and recommendations. The manuscript has been thoroughly revised based on their comments/feedback. Additionally, we have included a point-by-point response to address all the reviewers' comments. We honestly appreciate their contributions, which have significantly improved the quality of our manuscript.

Thank you for your attention.

Kind regards,

Sincerely,

Corresponding Author

Prof. Dr. Shahid Niaz Khan

Dean Faculty of Biological Sciences

Kohat University of Science and Technology, Kohat

**Novel Mutations in CYBB Gene Caused X-linked Chronic Granulomatous Disease in Pakistani Patients**

**Response to the Editor**

First, we sincerely appreciate and acknowledge the valuable suggestions and recommendations that have been recommended/suggested by the editor and reviewers (Reviewers 1 and 2). These suggestions have significantly contributed to the improvement of our manuscript. We would like to express our gratitude to the editor and reviewers for considering our first revised manuscript for possible publication. We have addressed each comment made by the reviewer point by point, as outlined below.

**Reviewers’ comments and Author response s**

**Reviewer reports:**

**Reviewer #1:**

**Comment 1:** The article evaluates patients with suspected chronic granulomatous disease. I believe that the most interesting aspect of the paper is describing patients from Pakistan (a country from which only few studies are available on inborn errors of immunity) and finding new possibly pathogenic variant for the gene CYBB. These two aspects should be clearly incorporated into the title.

Responses: We are grateful to Reviewer #1 for appreciating and providing us with insightful comments. We have taken their suggestions into consideration and changed the title accordingly.

**Comment 2:** However, in the current form, I think that the article is missing too many fundamental details and the article layout is not clear.

In particular:

- the authors adequately start with the clinical description of the cohort (this cohort includes patients with SUSPECTED CGD and NOT WITH CGD). Then, they adequately identify the five patients with pathological DHR test. But then only two patients were included in the paper (in addition this aspect is not clear from the abstract). I think that this approach is extremely confusing. Authors should mention the total patients evaluated, then report the ones who were "positive" for the clinical screening for CGD (SUSPECTED CGD), then report the pathological DHR and then discuss the genetic results on the entire cohort.

Response: We highly appreciate the valuable suggestions of the reviewer. We evaluated the capacity of neutrophils to produce H2O2 in a total of 17 patients. Among these patients, only five (P1, P2, P3, P4, and P5) displayed impaired H2O2 production. Consequently, we have now included and discussed the clinical presentations/results of these five patients.

**Comment 3:** As mentioned before, the result part of the abstract is totally unclear and should be reformulated.

Response: We are thankful for the valuable suggestions provided by the reviewer, and we have made changes to the entire abstract as per the recommendation of the reviewer.

**Comment 4:** Overall, I think that a complete rewriting of the results with changes as suggest above may be necessary.

Response: We are grateful for your comments, and we have thoroughly rewritten the entire result section, incorporating the necessary changes.

**Reviewer #2: The study is interesting. However, a few issues must be faced:**

**Comment 1:** The English must be extensively revised.

Response: We are thankful to Reviewer # 2 for appreciating our work and providing valuable suggestions. We have carefully edited the English as recommended.

**Comment 2:** The discussion should be shortened.

Response: We are grateful for the reviewer’s recommendation, and we have rephrased the entire discussion section of our manuscript and shortened it up to 683 words only, as per the suggestion.

**Comment 3:** The effective treatment with rhIFN must be proved using objective biomarkers.

Response: Thank you for your valuable suggestion. we have included the data regarding the in vitro treatment of monocyte-derived macrophages (MDMs) from gp91phox deficient patients with rhIFN-𝛾, which improved defective control of the proliferation of M. tuberculosis in MDMs from gp91phox functional deficient patients. Usually, treatment options for CGD are antibacterial prophylaxis (trimethoprim-sulfamethoxazole), antifungal prophylaxis (itraconazole), recombinant human interferon-gamma (rhIFN), and Hematopoietic stem cell transplant (HSCT) [1-6]. Accordingly, our CGD patients have been receiving antibacterial and antifungal prophylaxis. However, it is important to note that our CGD patients have not been receiving rhIFN-𝛾 as an immunoadjuvant nor undergoing HSCT. However, we conducted in vitro treatment of MDMs from gp91phox deficient patients with rhIFN-𝛾 and observed an improvement in their defective control of M. tuberculosis proliferation by MDMs. This finding is in accordance with previously reported studies [7, 8]. It is worth mentioning that further validation of our results regarding the in vitro treatment of rhIFN-𝛾 needs to be performed, particularly with a large sample size of MDMs from gp91phox functional deficient patients.

Regards,

Professor Dr. Shahid Niaz Khan

Dean Faculty of Biological Sciences

Kohat University of Science and Technology, Kohat
